# Delta-9 tetrahydrocannabinol (THC) inhibits lytic replication of gamma oncogenic herpesviruses *in vitro*

**DOI:** 10.1186/1741-7015-2-34

**Published:** 2004-09-15

**Authors:** Maria M Medveczky, Tracy A Sherwood, Thomas W Klein, Herman Friedman, Peter G Medveczky

**Affiliations:** 1 Department of Medical Microbiology and Immunology, MDC Box 10, University of South Florida, and the H. Lee Moffitt Cancer Center, 12901 Bruce B. Downs Blvd, Tampa, FL 33612-4799, USA

## Abstract

**Background:**

The major psychoactive cannabinoid compound of marijuana, delta-9 tetrahydrocannabinol (THC), has been shown to modulate immune responses and lymphocyte function. After primary infection the viral DNA genome of gamma herpesviruses persists in lymphoid cell nuclei in a latent episomal circular form. In response to extracellular signals, the latent virus can be activated, which leads to production of infectious virus progeny. Therefore, we evaluated the potential effects of THC on gamma herpesvirus replication.

**Methods:**

Tissue cultures infected with various gamma herpesviruses were cultured in the presence of increasing concentrations of THC and the amount of viral DNA or infectious virus yield was compared to those of control cultures. The effect of THC on Kaposi's Sarcoma Associated Herpesvirus (KSHV) and Epstein-Barr virus (EBV) replication was measured by the Gardella method and replication of herpesvirus saimiri (HVS) of monkeys, murine gamma herpesvirus 68 (MHV 68), and herpes simplex type 1 (HSV-1) was measured by yield reduction assays. Inhibition of the immediate early ORF 50 gene promoter activity was measured by the dual luciferase method.

**Results:**

Micromolar concentrations of THC inhibit KSHV and EBV reactivation in virus infected/immortalized B cells. THC also strongly inhibits lytic replication of MHV 68 and HVS *in vitro*. Importantly, concentrations of THC that inhibit virus replication of gamma herpesviruses have no effect on cell growth or HSV-1 replication, indicating selectivity. THC was shown to selectively inhibit the immediate early ORF 50 gene promoter of KSHV and MHV 68.

**Conclusions:**

THC specifically targets viral and/or cellular mechanisms required for replication and possibly shared by these gamma herpesviruses, and the endocannabinoid system is possibly involved in regulating gamma herpesvirus latency and lytic replication. The immediate early gene ORF 50 promoter activity was specifically inhibited by THC. These studies may also provide the foundation for the development of antiviral strategies utilizing non-psychoactive derivatives of THC.

## Background

The Kaposi's Sarcoma-Associated Herpesvirus (KSHV/HHV-8) is the likely cause of Kaposi's sarcoma in AIDS patients, aging individuals and organ transplant patients [for review, see [1]]. KSHV is also implicated in AIDS-associated primary effusion lymphoma (PEL) and a subset of cases of the lymphoproliferative disorder multicentric Castleman's disease. Epstein-Barr virus (EBV) belongs to the same group of herpesviruses and is also involved in human malignancies such as Burkitt's lymphoma, Hodgkin's disease, nasopharingeal carcinoma, and AIDS-associated lymphoma [for review, see [2]]. Related viruses such as herpesvirus saimiri (HVS) of monkeys and the murine gamma herpesvirus 68 (MHV-68) have been developed as animal models [3-7]. The DNA genomes of these oncogenic viruses persist in nuclei of lymphoid cells in a latent episomal circular form and a few of these cells can produce small amounts of infectious virus. In response to extracellular signals, the latent virus can be reactivated leading to production of more infectious virus progeny. This switch from latent to lytic infection is thought to be important in the pathogenesis of herpesviruses and the spread of infection within the organism and among individuals.

Reactivation of latent virus is often initiated by extracellular signals activating through receptors and various transcription factors. Transcription factors activate the promoter of the critical viral gene open reading frame 50 (ORF 50) of KSHV, HVS, MHV 68 or its homologue Rta of EBV [8-16]. The ORF 50 protein is also a transcription factor and further boosts production of its own mRNA [10,13,16]. An important function of ORF50/Rta is to activate early lytic genes [1,2] involved in DNA synthesis. After synthesis of early proteins, the process culminates with expression of late genes leading to production of virion components, virus assembly, release of progeny virus and cell death.

The major psychoactive cannabinoid compound of marijuana, Δ^9 ^tetrahydrocannabinol (THC), has been shown to modulate and primarily suppress immune responses against various pathogens [for review see [17,18]]. THC binds to and activates either one of the two cannabinoid receptors (CB1 and CB2) located on the surface of both brain and lymphoid cells [17-26]. CB1 and CB2 belong to the family of G protein-coupled receptors characterized by seven transmembrane loops interacting with the ligand on the outer surface of the cell. The receptors also contain an intracellular signaling domain. Several endogenous natural ligands of the CB receptor family termed endocannabinoids have been described. One example is anandamide, a lipid eicosanoid compound generated by the arachidonic acid pathway. The CB receptors are conserved through various vertebrate species including mammals and even fish. They have been shown in various tissues to modulate signaling and gene activation in response to short-lived endocannabinoid ligands or THC. However, THC has been also proposed to influence cellular function by other mechanisms due to its hydrophobic nature and likely association with lipid structures such as cell membranes [for review, see [17,18]]. This membrane-mediated effect is clearly less specific as all types of cells may be subject to it.

Since THC is an immune modulator we hypothesized that it may have an effect on gamma herpesvirus replication and/or latency. The data presented show that THC inhibits reactivation and lytic replication of these herpesviruses, possibly through inhibition of the ORF 50 promoter.

## Methods

### Tissue culture cell lines, virus, and THC

The KSHV positive primary effusion lymphoma cell line BCBL-1 isolated by Don Ganem and co-workers [27], and BC-3 isolated by Ethel Cesarman and co-workers [28], were obtained through the US National Institutes of Health AIDS Research and Reference Reagent Program (Rockville, MD, USA) and from the American Type Culture Collection (Manassas, VA, USA). The EBV positive P3HR1 cell line was from George Miller [29]. MHV 68 was obtained from Jeffrey Sample (St. Jude Children's Research Hospital, Memphis TN USA). NIH3T12 cells were from Samuel Speck (Yerkes Primate Research Center, Emory University, Atlanta, GA, USA). HVS strain 484 was isolated as described [30]. Owl monkey kidney (OMK) cells were from Danny Daniel (New England Research Primate Center, Southborough, MA, USA). THC was obtained from the National Institute on Drug Abuse, NIH (Bethesda, MD, USA). Three independent batches of synthetic THC were tested that gave very similar results. The purity of these THC preparations exceeded 99%.

### Antiviral assay based on the Gardella gel method

Cell suspensions were loaded in wells of a vertical agarose gel as described [31,32]. The cell layer was then overlaid with a lysis solution containing SDS and pronase resulting in gentle cell lysis and liberation of cellular and viral DNA. The gel was subjected to electrophoresis. Latent episomal DNA migrates much more slowly in these gels than linear replicating DNA [31,32]. After electrophoresis, Southern blotting was performed; DNA was transferred to nitrocellulose followed by hybridization with radiolabeled cloned viral DNA as described [32]. Radioactive images were analyzed and quantitated by a phosphoimager. The antiviral effect (IC50) was quantitated by comparing the values obtained from the episomal bands with those from linear bands as described [32].

### Virus yield reduction and cytotoxicity assays

NIH3T12 cells were infected with MHV 68, OMK cells with HVS, and both cell types with HSV-1 at a multiplicity of infection of 2 in the presence or absence of various concentrations of THC dissolved in DMSO. Control cultures were treated with DMSO. IC50 was determined by measuring virus titers in THC treated and control samples after one cycle of freeze-thawing of the cultures. Additional controls were uninfected cells grown in the presence of THC or DMSO. THC or DMSO was present throughout the experiment.

### Dual luciferase promoter assays

To evaluate the effect of THC on the KSHV ORF 50 promoter, a DNA fragment corresponding to the promoter region upstream of the mapped mRNAs [reference [9], nucleotides 70513–71513] was cloned into the basic firefly luciferase vector (Promega, Inc., Madison Wisconsin). To assay the effect of THC on the MHV 68 ORF 50 gene, a DNA fragment containing the 0.4 kb full length ORF 50 promoter (nucleotides 66242–66652) cloned upstream of the firefly luciferase was provided by Dr. Samuel Speck [46,47]. About 5 μg KSHV ORF 50 firefly plasmid DNA was transfected into BCBL-1 cells. About 5 μg MHV 68 ORF 50 firefly plasmid DNA was transfected into NIH312 cells. Control renilla luciferase expression vector (under the control of the CMV immediate early promoter) was included and co-transfected along with the ORF 50 reporter constructs. Cells were incubated for 48 h with either 5 μg/ml THC solution or an equal amount of DMSO. Luciferase activities were determined using a dual luminometer.

## Results

### THC inhibits KSHV and EBV DNA replication

**Figure 1:**
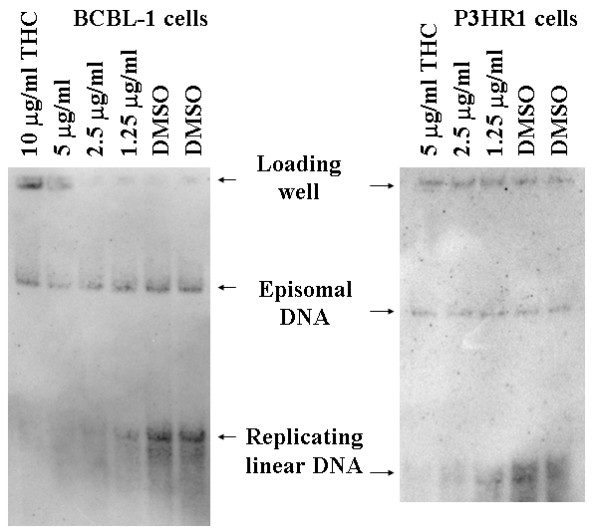
**THC Inhibits of KSHV and EBV linear DNA replication. **BCBL-1 or P3HR1 cell cultures were grown in standard RPMI/fetal calf serum medium. Cultures were supplemented with either various concentrations of THC dissolved in DMSO (as indicated) or with equivalent volumes of DMSO. After three days, cells were analyzed for latent episomal and lytic linear viral genomes as described [31,32]. Briefly, 10^6 ^cells were loaded in wells of a vertical agarose gel then overlaid with a lysis solution containing SDS and pronase, resulting in gentle cell lysis and liberation of cellular and viral DNA. The gel was subjected to electrophoresis. After electrophoresis, Southern blotting was performed and DNA was transferred to nitrocellulose followed by hybridization with radiolabeled overlapping cosmid clone probes of KSHV, representing the entire genome, as described [32] **(*left panel*) **or with radiolabeled cloned Bam W fragment of EBV **(*right panel*)**. Latent episomal DNA migrates much more slowly in these gels than linear replicating DNA [31,32]. Arrows on the autoradiograms indicate the position of both species of DNA.

To evaluate the effect of THC on KSHV replication, we selected BCBL-1 and BC-3 lymphoblastoid cells because these cultures spontaneously produce small amounts of virus, and we used a previously published antiviral drug testing protocol [32] to determine whether THC induces or inhibits virus replication. Dead cells from BCBL-1 or BC-3 lymphoblastoid cell cultures were removed by Ficoll gradients. Cells were cultured in RPMI 1640 growth medium containing 10% serum for 3 days in the presence or absence of various concentrations of THC (dissolved in DMSO). Control cells were cultured in the presence of 0.1% DMSO (this concentration of DMSO was also used in THC treated cultures). Cells were suspended and loaded in wells of a vertical agarose gel, then overlaid with a lysis buffer to remove proteins from DNA. After lysis, slowly migrating episomal DNA representing the resident latent viral genome, and fast migrating linear DNA representing lytic virus replication, were separated by electrophoresis. After electrophoresis, viral DNA was visualized by Southern blotting. Figure 1, left panel shows that control DMSO-treated BCBL-1 cells spontaneously produce a small amount of linear DNA, indicating lytic replication/reactivation in a subpopulation of the cells. Various concentrations of THC showed concentration-dependent inhibition of linear but not episomal KSHV DNA. These data were reproducible and similar observations were made in five independent experiments. Based on these experiments we calculated the 50% inhibitory concentration (IC_50_) of THC at 1 μg/ml or about 3.3 μM.

To evaluate whether another KSHV strain is also susceptible to THC, the BC-3 cell line was also tested. In these cells, THC had a similar inhibitory effect on KSHV reactivation as observed with BCBL-1 cells (not shown).

Two experiments analogous to the one described for KSHV were performed with the EBV transformed B cell line P3HR1. P3HR1 cell cultures were grown in the presence of the phorbol ester TPA, because EBV did not reactivate spontaneously and TPA was essential to induce lytic EBV replication. The cultures were also supplemented with either various concentrations of THC dissolved in DMSO or with equivalent volumes of DMSO. Fig. 1, right panel, shows a representative experiment. The autoradiogram indicates that THC inhibited EBV linear DNA synthesis. Based on quantitative analysis of the radioactive bands and extrapolation of data, the 50% inhibitory concentration (IC_50_) for THC was estimated at around 0.9 μg/ml or about 3 μM.

### THC inhibits MHV 68 and HVS lytic replication in monolayer cells

**Figure 2:**
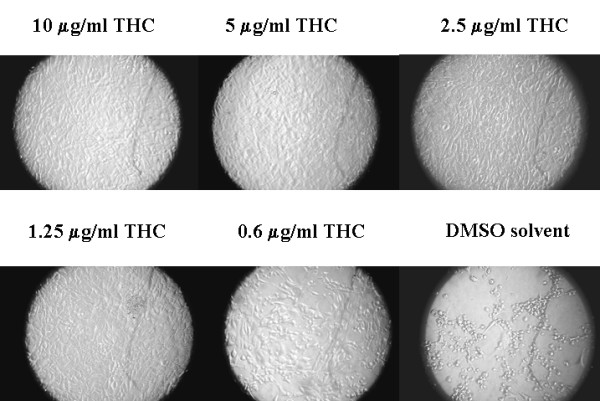
**THC inhibits MHV 68 cytopathic effect in NIH3T12 cells**. Photograph of NIH3T12 infected with MHV 68 at a multiplicity of 2. Plates were cultured for 48 h in the presence or absence of various concentrations of THC (0.625–10 μg/ml) or equivalent volumes of DMSO.

We examined the effects of THC on the replication of MHV68 and HVS, two rhadinoviruses related to KSHV and to lesser extent to EBV. These viruses can infect monolayer cells and are suitable for testing the effects of compounds on lytic virus replication by virus yield reduction assays, a more widely used test for evaluating antiviral drugs. NIH3T12 cells were infected with MHV68 at a multiplicity of infection of 2 in the presence or absence of various concentrations of THC dissolved in DMSO. Control cultures were treated with DMSO. THC or DMSO was present throughout the experiment. Cell cultures were incubated for 48 h. As shown in Figure 2, a typical full cytopathic effect of MHV 68 was observed in the control infected cell culture treated with DMSO; most adherent cells become detached from the plate, and remaining loosely adhered cells were round and denser than uninfected controls. However, when infected cells were cultured in the presence of 1.25 μg/ml or higher concentrations of THC, they remained indistinguishable from uninfected control cells and remained adhered to the plate. The effect of 0.6 μg/ml of THC was intermediate between uninfected cells and the full cytopathic effect. These results indicated a protective effect of THC against destruction of host cells by the virus and suggested that THC may inhibit MHV 68 replication. Similar results were obtained with HVS in owl monkey kidney cells (not shown).

**Figure 3:**
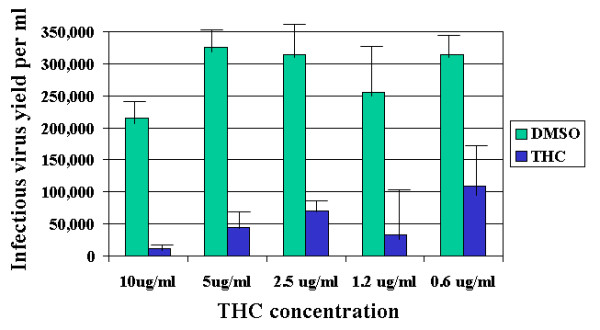
**THC inhibits MHV 68 virus yield in NIH3T12 cells**. NIH3T12 monolayer cultures propagated in 24-well plates were infected and cultured in the presence of various concentrations of THC or equivalent DMSO. Forty-eight hours after infection, control cultures showed complete cytopathic effect and destruction of cells. To liberate cell-associated virus, cultures were subjected to a cycle of freeze-thawing; cells were homogenized and virus was titrated by end point dilution in 96-well plates.

To quantitatively determine antiviral effects of THC, yield reduction assays were performed. NIH3T12 cell cultures were infected with MHV 68 and incubated with THC or DMSO dilutions for 48 h. Cell-associated virus was liberated by freeze-thawing. After low speed centrifugation, virus titer was determined in the culture supernatants. Figure 3 shows that virus yield (expressed as infectious units of virus per ml) was highly significantly inhibited by THC. Inhibition of virus yield was over 300-fold at 10 μg/ml THC. The 50% inhibitory concentration (IC_50_) was estimated at around 0.6 μg/ml, equivalent to about 1.9 μM. Similar results were obtained from two independent experiments. Similar and reproducible results were obtained with HVS in monkey kidney cells (not shown).

### THC is not cytotoxic to murine NIH3T12 or owl monkey kidney (OMK) cells

**Figure 4:**
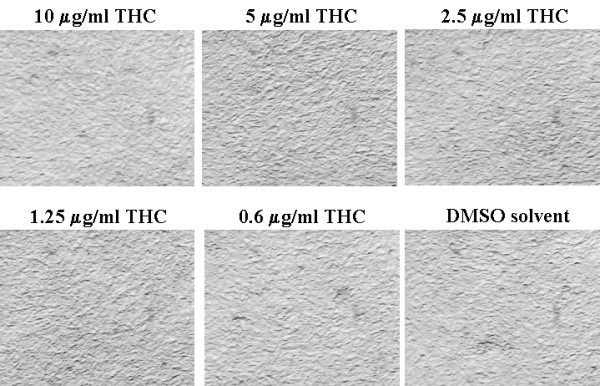
**THC is not toxic to NIH3T12 cells**. NIH3T12 cells seeded at about 1/4th of confluency were cultured for 48 h in the presence of various concentrations of THC (0.625–10 μg/ml) and photographed.

Because the observed effects might be due to a non-specific toxic effect of THC, we tested whether THC alters cell division or morphology in NIH3T12 and owl monkey kidney (OMK) cells. Monolayers of these cells were prepared at about 25% confluency and cultured for 2 days in the presence of THC concentrations ranging 0.6–10 μg/ml. A series of photographs (Figure 4) show that THC treated cultures were indistinguishable from control cultures, formed confluent monolayers, and showed no altered morphology. Owl monkey kidney (OMK) cells also showed no toxicity in an analogous experiment (not shown).

The effect of higher THC concentrations on cell division was also determined. Exponentially growing BCBL-1 or NIH12 cells were cultured for two days in the presence of various concentrations of THC or DMSO and cell counts were determined. The 50% cell division inhibitory concentration of THC for BCBL-1 cells was around 33 μM. NIH3T12 cells were less sensitive and the 50% inhibitory concentration was around 99 μM (not shown).

### THC has no comparable effect on HSV-1 lytic replication

**Figure 5:**
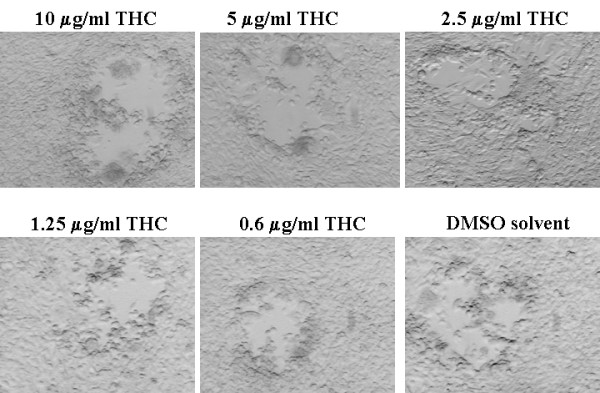
**THC has no significant effect on plaque formation of HSV-1 in NIH3T12 cells**. NIH3T12 monolayer cultures propagated in 6-well plates were infected with about 100 infectious units of HSV-1 per plate and cultured in the presence of various concentrations of THC or equivalent DMSO solvent. Photographs were taken four days after infection.

We next examined whether or not the THC inhibitory effect can be observed against other herpesviruses and tested whether drug treatment suppresses the replication of the alpha herpesvirus, HSV-1. Monolayers of NIH3T12 were prepared, infected with about 100 infectious units of HSV-1, and cultured for 3 days in the presence of THC concentrations ranging 0.6–10 μg/ml. A series of photographs shows (Figure 5) that typical plaques developed in THC treated cultures, indistinguishable from those in control cultures. HSV-1 replication in OMK was also unaffected by THC in an analogous experiment (not shown).

**Figure 6:**
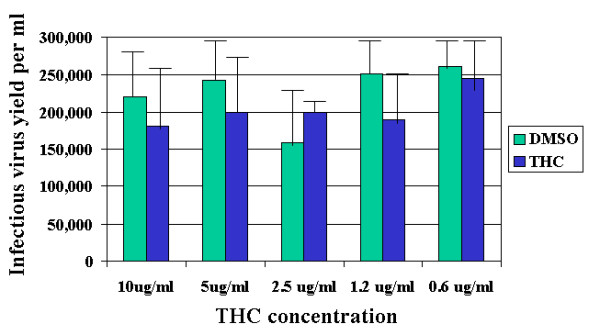
**THC has no significant effect on virus yield of HSV-1 in NIH3T12 cells**. NIH3T12 monolayer cultures propagated in 24-well plates were infected with HSV-1 at a multiplicity of 2 and cultured in the presence of various concentrations of THC or equivalent DMSO. Twenty-four hours after infection, all cultures showed complete cytopathic effect and destruction of cells. To liberate cell-associated virus, cultures were subjected to a cycle of freeze-thawing; cells were homogenized and virus was titrated.

We also examined the effects of THC on production of HSV-1 virus yield in NIH3T12 cells. Cells were infected with HSV-1 at a multiplicity of infection of 2 in the presence or absence of various concentrations of THC dissolved in DMSO. Control cultures were treated with DMSO. THC or DMSO was present throughout the experiment. Cell cultures were incubated for 24 h and cell-associated virus was liberated by freeze-thawing. After low speed centrifugation the HSV-1 titer was determined in the culture supernatants. Figure 6 shows that THC had no significant inhibitory effect on replication of HSV-1 in NIH3T12 cells.

### THC inhibits the ORF 50 promoter

To evaluate the effect of THC on the MHV 68 and KSHV ORF 50 promoters, luciferase reporter assays were performed. To assay the effect of THC on the MHV 68 gene, a DNA construct published by the Speck laboratory [46,47] containing the 0.5 kb full length ORF 50 promoter cloned upstream of the firefly luciferase was transfected into NIH312 cells. Control renilla luciferase expression vector (under the control of the CMV immediate early promoter) was included and co-transfected with the ORF 50 reporter. Cells were incubated for 48 h with either 5 μg/ml THC or equal amount of DMSO. Luciferase activities were determined using a dual luminometer. The results show (Table 1) that 5 μg/ml THC suppressed the ORF 50 promoter about 7.4 fold. In contrast, the control CMV promoter activity was only reduced by 35%. Similar results were obtained with the KSHV ORF 50 promoter construct containing 1 kb of the promoter sequence. In the transfected BCBL-1 cells, 5 μg/ml THC inhibited the ORF 50-driven luciferase activity almost 4-fold. Interestingly, the co-transfected CMV promoter was slightly stimulated by THC in these cells. These results were obtained from three independent transfection experiments for both the MHV 68 and KSHV promoter assays.

### Cannabinoid receptor antagonists can reverse the inhibitory effect of THC on KSHV replication

**Figure 7:**
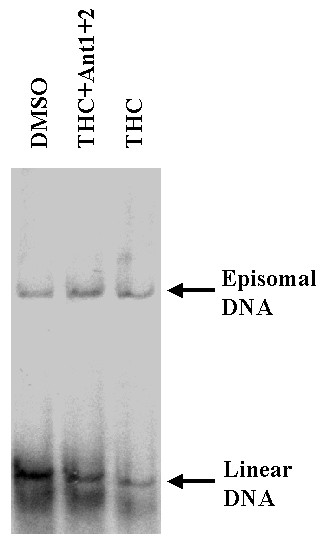
**Effect of receptor antagonists on THC mediated suppression of KSHV reactivation. **BCBL-1 cells were cultured in the presence of 1.25 μg/ml THC (lane THC), 1.25 μg/ml THC plus 5× molar excess of SR141716A and SR144528 for 72 h (lane Anti1+2), or in medium containing equivalent amount of solvent (DMSO). Cells were analyzed for episomal control and linear replicating DNA as described in Figure 1.

To investigate whether the observed antiviral effects of THC are mediated through the cannabinoid receptors, two antagonists of THC, SR141716A (which acts on CB1) and SR144528 (which inhibits CB2; a gift from Dr. Pierre Casallas, Sanofi Recherche), were tested in the standard BCBL-1 KSHV reactivation assay (Figure 7). BCBL-1 cells were incubated with or without THC and in the presence of these compounds. The THC inhibition of KSHV DNA synthesis was not reversed by treatment with a single receptor antagonist (not shown). The antagonists had no effect on spontaneous KSHV reactivation. However, Figure 7 shows that they reversed the inhibition of linear DNA synthesis by 1.25 μg/ml THC.

## Discussion

To summarize the antiviral effects of THC and to compare THC with well-characterized antiviral drugs, we compiled data from the literature as well as from our own experimental results. Table 2 shows the 50% inhibitory concentrations (IC_50_) of four known antiviral drugs and of THC. Data on the antiviral effects of acyclovir, PFA and ganciclovir on KSHV were obtained from our Gardella gel assays [32]. Usherwood et al. [33] also used a Gardella gel-based assay to determine the effect of acyclovir against MHV 68 in transformed B cell line S11. The data regarding the effects of four antiviral drugs against MHV 68 and on cell division are from the work of Neyts and De Clercq [34] using standard lytic virus replication inhibition assays on monolayers. The THC antiviral results are from the data described in this paper (Gardella gels for KSHV using BCBL-1 cells and NIH12 antiviral assays for MHV 68). These data suggest that THC is a potent and selective antiviral agent against KSHV comparable with some well-characterized anti-herpesvirus compounds. THC is even more potent and selective against MHV 68 than acyclovir, ganciclovir and foscarnet. Cidofovir appears to be most potent in these *in vitro *experiments; however, this drug is known to cause serious side effects and is toxic to the kidney in humans.

As outlined earlier, THC can modulate and inhibit the activation of immune cells, so it is not entirely surprising that it can down-regulate the reactivation of viruses residing in lymphocytes, as shown by the data. However, the antiviral effect of THC is not cell specific, since MHV 68 and HVS replication was also strongly inhibited by THC in NIH3T12 or owl monkey kidney monolayer cell cultures. This observation suggests that THC either directly or indirectly targets a viral gene shared by these herpesviruses.

The data presented in Figure 7 suggest that THC may inhibit KSHV replication through the cannabinoid receptors. When BCBL-1 cells were treated with THC the receptor antagonists partially reversed this effect, suggesting a role for the CB receptors expressed by BCBL-1 cells. However, more studies are required to evaluate whether CB1, CB2 or both receptors are involved. As discussed in the Introduction, ORF 50/Rta is a critical gene for both reactivation of latent virus and lytic replication in monolayers. Interestingly, ORF 50/Rta activation involves cAMP signaling [12,35]. In contrast, cannabinoid receptor binding has been shown to down-regulate the level of activated CREB through a decrease in cyclic AMP synthesis [17,18,26,36]. Therefore, one possible explanation is that THC inhibits cAMP signaling, leading to decrease of ORF 50/Rta-mediated transcription and block of virus replication. This antiviral mechanism of THC is supported by our data. Luciferase reporter assays showed that in the presence of THC, initiation of transcription of ORF 50 mRNA in both KSHV and MHV 68 is markedly reduced (4-fold and 7.4-fold, respectively) as compared with the CMV immediate early promoter. These data suggest selective inhibition of the ORF 50 promoter of MHV 68 and KSHV by THC. However, it is also anticipated that THC may also block other cellular and viral genes,, as this drug has been shown to cause a wide range of changes in lymphocyte gene expression [17,18].

## Conclusions

Early studies have attempted to evaluate whether THC has effects on HSV-1 and HSV-2 replication [37-44]. Most of these studies concluded that THC directly or indirectly enhances replication/reactivation of these viruses, although Lancz et al. [42] showed that a very high concentration (330 μM) decreases the infectivity of virions. Our data presented in this paper show no effect of THC on HSV replication at lower concentrations. To resolve these conflicting observations, investigation of this issue should continue in the light of new advances in herpesvirus molecular biology and cannabinoid research.

Interestingly, statistical analysis indicates a lower incidence of Kaposi's sarcoma in HIV positive women using non-intravenous drugs [45]. About 5.4% of HIV positive women with no drug use developed KS, whereas none of the 47 women in this study who only used marijuana suffered from KS [ref. [45], and James Goedert, personal communication]. This report, however, involved relatively few individuals so further analysis of a larger cohort is warranted.

We believe that studies on cannabinoids and herpesviruses are important to continue because there are obvious potential benefits. Better understanding may lead to the development of specific non-psychoactive drugs that may inhibit reactivation of oncogenic herpesviruses.

## Competing interests

None declared.

## Authors' contributions

MMM carried out most of these studies. TAS carried out some of the luciferase assays. TWK and HF participated in the design of the study. PGM conceived the study and participated in its design and overall coordination. All authors read and approved the final manuscript.

## Pre-publication history

The pre-publication history for this paper can be accessed here:



## Figures and Tables

**Table 1:** Relative luciferase activity of extracts from control and THC treated cells. NIH 3T3 cells co-transfected with MHV 68 ORF 50 plus CMV control (top rows of the table). BCBL-1 cells were co-transfected with the KSHV ORF 50 plus CMV control (bottom rows of the table). Data represent averages from three independent co-transfection experiments.

**Treatment of NIH 3T3 cells**	**MHV 68 ORF 50 promoter (firefly luciferase)**	**CMV renilla luciferase**
DMSO	100% +/- 14%	100% +/- 11%
5 μg/ml THC	13.5% +/- 1.5%	65.8% +/- 9%
**Treatment of BCBL-1 cells**	**KSHV ORF 50 (firefly luciferase)**	**CMV renilla luciferase**

DMSO	100% +/- 14%	100% +/- 3%
5 μg/ml THC	28% +/- 13%	128% +/- 3%

**Table 2:** Comparison of 50% antiviral and cell division inhibitory concentrations (IC_50_) and selectivity (viral versus cellular IC_50_) of selected antiviral drugs and THC

**Inhibitor compound**	**KSHV IC_50 _(μM)**	**Cellular IC_50 _(μM)**	**Selectivity index (viral versus cellular IC_50_)**
Acyclovir	75 [ref. 32]	Not done	
Ganciclovir	5.1 [ref. 32]	Not done	
Foscarnet	97 [ref. 32]	Not done	
Cidofovir	0.05 [ref. 32]	Not done	
THC	3.3 (this work)	33 (this work)	10 (this work)
	**MHV 68 IC_50 _(μM)**		
Acyclovir	6 [ref. 34] 100 [ref. 33]	182 [ref. 34]	30 [ref. 34]
Ganciclovir	28 [ref. 34]	108 [ref. 34]	3.7 [ref. 34]
Foscarnet	120 [ref. 34]	1413 [ref. 34]	11 [ref. 34]
Cidofovir	0.08 [ref. 34]	78 [ref. 34]	10,000 [ref. 34]
THC	1.9 (this work)	99 (this work)	52 (this work)
